# A path toward self-acceptance: The mediating effects of body image between self-love, self-compassion, and self-acceptance in people with obesity

**DOI:** 10.1371/journal.pmen.0000609

**Published:** 2026-05-19

**Authors:** Rida Yanna Primanita, Aufizzahra As Syafiyah, Alfian Yanda Putra

**Affiliations:** 1 Department of Psychology, Faculty of Psychology and Health, Universitas Negeri Padang, Padang, West Sumatra, Indonesia; 2 Sahabat Indonesia Center for Psychological Services, Bukittinggi, West Sumatra, Indonesia; National University of Singapore, SINGAPORE

## Abstract

Obesity is a significant global health challenge often accompanied by weight stigma and psychological distress. This study aimed to examine the structural relationships between self-love, self-compassion, body image, and self-acceptance among adults with obesity in Indonesia. A cross-sectional online survey was conducted involving 200 adults with obesity (Body Mass Index > 23 kg/m²). The participants consisted of 71% women and 29% men, all within the age range of 18–40 years. Data were collected using self-report measures and analyzed using path analysis with bias-corrected bootstrapping (5,000 resamples) to estimate direct and indirect effects. The findings revealed that self-compassion was the strongest positive predictor of self-acceptance. Contrary to expectations, neither self-love nor body image significantly predicted self-acceptance directly. Furthermore, body image did not mediate the relationships between self-love or self-compassion and self-acceptance. These results suggest that for adults with obesity in Indonesia, self-acceptance is predominantly driven by internal emotion-regulation mechanisms (self-compassion) rather than by body evaluations or motivational self-love. Psychological interventions should prioritize fostering self-compassion to enhance well-being in this population.

## Introduction

The prevalence of obesity has exhibited a worrisome upward trend both globally and nationally over the past three decades. Recent estimates indicate that over 1.9 billion adults worldwide are overweight, with more than 650 million living with obesity—representing a threefold increase since 1975 [1]. If this trajectory persists, projections suggest that by 2030, more than half of the global adult population will be categorized as overweight or obese [1,2]. Furthermore, updated forecasting models indicate that by 2050, the number of adults with overweight and obesity could reach approximately 3.8 billion [3].

A comparable scenario is observed in Indonesia, where data from the 2018 Basic Health Research (Riskesdas) survey suggest a substantial increase in obesity prevalence in urban regions, rising from 23.0% in 2007 to 50.1% in 2018, concurrent with an escalation in central obesity from 28.0% to 57.2% during the same period [4]. Obesity is recognized as having multidimensional consequences, encompassing physical health risks such as hypertension, type 2 diabetes, and cardiovascular disease [5–7]. As indicated by the extant literature, the psychosocial impacts of the condition include stigma, discrimination, diminished self-esteem, and reduced quality of life [8,9]. These conditions underscore the necessity of directing attention not only to the medical aspects but also to the psychological dimensions in developing comprehensive strategies to address obesity.

Self-acceptance, defined as an individual’s capacity to fully embrace themselves, including their physical condition, is a critical component of psychological well-being among adults with obesity [10]. Research consistently demonstrates that individuals with obesity exhibit lower levels of self-acceptance compared to those with normal body weight. This discrepancy is largely driven by body dissatisfaction, shame, and self-criticism [11]. This vulnerability is frequently exacerbated by cultural pressures and media portrayals of the “ideal” body, which serve to reinforce self-rejection and diminish self-esteem. The internalization of shame, in turn, is further compounded by social stigma and discrimination, thereby undermining overall quality of life [12,13]. Such negative self-perceptions are evident across the lifespan, ranging from parental teasing in childhood to significantly lower self-esteem reported by adults with obesity across genders, as well as poorer body image among individuals enrolled in weight management programs [14]. The findings of this study collectively indicate that low self-acceptance is not merely a psychological consequence of obesity, but also a substantial barrier to effective weight management and overall well-being.

At the same time, self-acceptance plays a pivotal role in sustaining psychological well-being among adults with obesity, as it is positively associated with life satisfaction and overall mental health [15]. Higher levels of self-acceptance have been shown to enhance an individual’s capacity to manage stress and maintain positive interpersonal relationships [16]. Conversely, persistently low self-acceptance has been demonstrated to increase vulnerability to depression, anxiety, and social withdrawal driven by stigma [17]. Within the paradigm of positive psychology, self-acceptance is recognized as a key indicator of optimal functioning, fostering personal growth, autonomy, and life balance [18].

The development of self-acceptance among adults with obesity is not a static condition that emerges automatically; rather, it is the outcome of a complex interplay of protective internal factors. According to the positive psychology literature, two key predictors that substantially contribute to the development of self-acceptance have been identified: self-love and self-compassion [19,20]. Despite the frequent interchangeability of these three constructs in non-specialist discourse, theoretical frameworks distinctly differentiate them, delineating their representation of qualitatively disparate psychological processes operating at varying levels of one’s relationship with the self. Consequently, a rigorous conceptual differentiation is imperative to elucidate the unique pathways through which each construct is associated with the psychological well-being of individuals with excess body weight.

Fundamentally, self-love is an affective and motivational construct that reflects an orientation toward valuing oneself and prioritizing personal well-being unconditionally [20,21]. Self-love has been demonstrated to encourage individuals to perceive themselves as inherently worthy of love, regardless of physical imperfections or external failures. This, in turn, has been shown to be positively related to body appreciation [22]. In contrast, self-compassion operates predominantly as an active emotion-regulation strategy in circumstances involving suffering or failure. According to Neff’s [19] conceptualization, the practice of self-compassion involves a compassionate stance toward oneself, manifested through three core components: self-kindness, a sense of common humanity, and mindfulness. Conversely, self-acceptance is regarded as a more stable and comprehensive psychological outcome, characterized by an individual’s cognitive-evaluative stance to integrate all aspects of their self, independent of external validation [23].

Although self-compassion and self-acceptance are frequently discussed as related constructs, accumulating empirical evidence demonstrates that they represent distinct psychological processes with separable mechanisms. Factor-analytic studies provide robust psychometric evidence for this distinction, consistently identifying them as separate factors rather than a unified dimension [24–26]. Correlational and intervention research further supports their discriminant validity, demonstrating differential predictive patterns: self-compassion operates primarily as an active coping strategy that mitigates distress (e.g., anxiety, guilt, and self-blame), whereas unconditional self-acceptance functions as a stable baseline of self-worth that shows smaller associations with acute distress regulation [27,28]. Mediation analyses further clarify this temporal sequence, suggesting that compassionate self-responding facilitates the state of self-acceptance, which subsequently supports well-being [29]. Thus, while both contribute uniquely to psychological health, self-compassion represents the active regulatory process, and self-acceptance represents the stable evaluative outcome.

Within the specific context of obesity, self-love and self-compassion operate synergistically yet through distinct mechanisms in fostering self-acceptance. Self-love serves as a motivational foundation that instills the internalized belief that “I am worthy of care and respect”. This belief serves as a protective factor against the internalization of weight bias [30]. When individuals with obesity exhibit high levels of self-love, they are more likely to engage in health-promoting behaviors stemming from a sense of respect for their bodies rather than body-related self-hatred. Conversely, the practice of self-compassion operates as a psychological cushion, providing emotional resilience when confronted with social stigmatization or challenges in weight management. Empirical evidence demonstrates that self-compassion significantly reduces body shame and body dissatisfaction by disrupting cycles of harsh self-criticism, thereby enabling individuals to maintain self-acceptance even when their physical condition does not conform to prevailing social ideals [31–33].

Thus, these two exogenous variables contribute to the endogenous outcome (self-acceptance) through complementary pathways: self-love provides a foundation of positive self-affirmation, whereas self-compassion supplies resilience-related skills for coping with adversity. The integration of these two aspects is particularly crucial within collectivistic cultural contexts, such as in Indonesia. In such contexts, overt expressions of self-love may be constrained by norms of modesty. Conversely, self-compassion, being closely aligned with communal values of empathy and interconnectedness, may serve as a culturally congruent and powerful alternative pathway toward self-acceptance. Consequently, examining self-love and self-compassion concurrently provides a more comprehensive understanding of how internal psychological resources can be mobilized to enhance self-acceptance among adults with obesity.

Moreover, the present study positions body image as a crucial mediating mechanism that bridges the associations of self-love and self-compassion with self-acceptance. Body image is conceptualized as a multidimensional construct encompassing individuals’ perceptions, thoughts, feelings, and behaviors toward their own bodies [34]. Within populations with obesity, these body-related evaluations are frequently distorted by internalized weight bias, consistently associating with lower self-esteem and diminished psychological well-being [35,36]. The theoretical rationale for conceptualizing body image as a mediator, rather than merely as a parallel outcome variable, is rooted in the Cognitive–Behavioral Model of Body Image [34]. Within this theoretical framework, body image functions as a proximal cognitive and affective evaluation that directly relates to global self-appraisals.

Conversely, self-love and self-compassion are conceptualized as more distal psychological resources that influence how individuals interpret their physical appearance under social pressure. Specifically, self-love provides a foundation of unconditional self-worth that protects individuals from appearance-based self-evaluations, whereas self-compassion attenuates the emotional impact of self-criticism through mechanisms of self-kindness and common humanity [19,33]. As these internal resources increase, adults with obesity are better equipped to cultivate positive body appreciation and reduce body shame [37,38]. Subsequent improvements within this specific domain (body image) function as a “cognitive-affective filter,” thereby positively correlating with global self-acceptance. This proposition aligns with the principle of bottom-up processing in self-concept formation, whereby acceptance of physical attributes that previously constituted sources of distress represents a critical prerequisite for achieving a coherent and integrated sense of self. Consequently, body image operates as a potential bridging mechanism that elucidates how positive self-directed attitudes relate to stable self-acceptance.

Despite the extensive documentation of the protective roles of self-love and self-compassion in the global literature, there are substantial empirical gaps regarding the operation of these mechanisms in non-Western contexts. The prevailing influence of findings derived from Western, Educated, Industrialized, Rich, and Democratic (WEIRD) societies imposes limitations on the ecological validity of prevailing psychological models when applied to cultures characterized by pronounced collectivistic orientations [39]. Within the sociocultural context of Indonesia, the concept of the self is not perceived as autonomous but rather as interdependent, influenced significantly by relational harmony and group-based expectations [40,41]. In contrast to individualistic contexts, where obesity stigma often manifests through formalized discrimination, in Indonesia, stigma is frequently expressed in more subtle and culturally specific forms, such as “weight-related teasing” or fat-shaming embedded within familial interactions or everyday social exchanges. Despite their common perception as innocuous jest, these practices can have profound psychological ramifications [42,43].

Although cultural dimensions were not empirically measured in the present cross-sectional design, this study employs culture as a critical conceptual and interpretative lens for understanding the dynamics among the focal constructs. This approach provides a more nuanced understanding of how self-love and self-compassion function not only as internal emotion-regulation resources but also as buffers against culturally embedded pressures toward social conformity. This contributes a novel perspective to the body image literature, which has been predominantly shaped by Western bias.

The present study aims to test an integrative model that explicates the structural relationships underlying self-acceptance among adults with obesity in Indonesia. Built on the theoretical foundations and literature gaps outlined above, this study examines the associations between self-love, self-compassion, and self-acceptance, with body image serving as a key mediating variable. This approach is designed to yield a comprehensive and culturally sensitive understanding of these processes within the local context. Beyond its contribution to the cross-cultural psychology literature, it is anticipated that the findings will provide an empirical foundation for the development of targeted clinical interventions.

The study proposes a conceptual framework to investigate seven primary hypotheses. Acknowledging the cross-sectional nature of the data, the proposed paths represent structural associations. It is hypothesized that self-love and self-compassion will positively predict self-acceptance (H1 and H2). It is also hypothesized that a more positive body image will be positively associated with self-acceptance (H3). Furthermore, it is expected that self-love and self-compassion will positively correlate with a more positive body image (H4 and H5). Finally, it is hypothesized that body image will mediate the indirect relationships of self-love and self-compassion with self-acceptance (H6 and H7).

## Materials and methods

### Ethics statement

This study was conducted in strict accordance with ethical principles for research involving human participants. The research protocol was reviewed and approved by the Research Ethics Committee of Padang State University (Approval No. 102/KEPK-UNP/12/2025; December 10, 2025). All participants provided informed consent prior to participation. For the online survey, an electronic informed consent page was presented at the beginning of the questionnaire, which explained the study objectives, voluntary nature of participation, confidentiality of responses, and participants’ rights. Consent was obtained by requiring participants to indicate their agreement before accessing the survey items. All data were collected anonymously, and no personally identifiable information was recorded.

### Research design

The present study employed a quantitative correlational design with a cross-sectional approach to examine the structural relationships between self-love, self-compassion, and self-acceptance, as well as the mediating role of body image among adults with obesity in Indonesia [44]. The cross-sectional design was selected to capture data at a single point in time, with data collection conducted between January and May of 2025. The survey was administered online using self-administered questionnaires hosted on Google Forms and distributed via digital platforms to participants across Indonesia [45]. In order to guarantee data integrity and prevent the occurrence of multiple submissions, a rigorous data quality protocol was implemented. The process entailed configuring platform settings to restrict response frequency and conducting a manual screening of the dataset to identify duplicate entries based on identical demographic profiles (e.g., age, exact BMI, domicile) and submission timestamps. After thorough examination, it was determined that the final dataset did not contain any duplicate cases. The ethical principles were scrupulously observed during the course of the study. The online interface incorporated an informed consent page that delineated the objectives of the study, the voluntary nature of participation, and the confidentiality assurances. Participants were requested to provide implied consent by checking an agreement box before proceeding.

### Participants

Participants were recruited using a non-probability purposive sampling technique based on the following inclusion criteria. The study’s inclusion criteria are as follows:

1. Subjects aged 18–40 years old;
2. Body Mass Index (BMI > 23 kg/m²), indicating overweight or obesity according to the Asia-Pacific classification [46];
3. Residing in Indonesia; and
4. Willingness to participate voluntarily.

As data collection was conducted online, anthropometric measurements (height and weight) were self-reported. Participants were instructed to report their current weight in kilograms and height in centimeters, with the understanding that they would provide an accurate assessment of their current physical state. The researchers subsequently calculated the body mass index (BMI) of the participants using the standard formula (kg/m²). The BMI was calculated with the intention of determining eligibility, with a cutoff of >23 kg/m² being applied to classify obesity according to the Asia-Pacific guidelines.

The final sample consisted of 200 respondents, which was deemed adequate for Structural Equation Modeling (SEM). It is well-established that a sample size of over 200 ensures stable and reliable parameter estimation [47]. The participants represented diverse geographic regions of Indonesia, including western, central, and eastern regions, thereby reflecting a broad sociocultural range.

### Instruments

The research questionnaire was composed of two distinct sections. The initial section of the study gathered demographic information, which functioned as control variables. This included gender, age, marital status, self-reported height, and self-reported weight. These variables were used to calculate the Body Mass Index (BMI). The second section of the study evaluated the four core study variables: self-love (X1), self-compassion (X2), body image (M), and self-acceptance (Y).

The assessment of the four core variables utilized established instruments whose construct validity had been rigorously confirmed in their respective prior validation studies. First, the construct of self-love was measured using an established scale developed by Rahmadaini et al. [48], which was grounded in the theoretical framework proposed by Xue et al. [20]. It is important to clarify that this measure was not newly developed for the current study; rather, it is a previously validated instrument adopted to ensure conceptual alignment with the study’s framework. Second, self-compassion was assessed using the scale adapted by Rahmi [49], with the underlying theoretical framework being Neff’s [19]. Third, the measurement of body image was performed using Annisa’s [50] adaptation of Cash and Smolak’s [34] multidimensional framework. Finally, the assessment of self-acceptance was conducted using a scale constructed based on Jersild’s [10] framework.

In the present study, the internal consistency of these instruments was re-verified, yielding robust reliability coefficients for self-love (Cronbach’s α = 0.907), self-compassion (α = 0.851), body image (α = 0.912), and self-acceptance (α = 0.920). Additionally, to eliminate the possibility of measurement redundancy between closely related constructs, a correlation analysis revealed a very weak, non-significant association between self-love and self-compassion (ρ = 0.056, p > 0.05). This finding supports their conceptual distinction.

### Data analysis

The data were analyzed using JASP software (version 0.95). The analytical procedure commenced with the implementation of descriptive statistics, encompassing the mean, median, interquartile range, standard deviation, minimum, maximum, skewness, and kurtosis. The assessment of normality was conducted through the implementation of the Shapiro–Wilk test, complemented by an examination of skewness and kurtosis values. Given the non-normal distribution of the data, initial bivariate associations were examined using Spearman’s rho correlations [47]. The reliability of the measurement instruments was evaluated using Cronbach’s alpha to ensure adequate internal consistency.

Furthermore, to address potential concerns regarding conceptual overlap and measurement redundancy between closely related constructs (particularly self-compassion and self-acceptance) collinearity diagnostics were conducted prior to structural testing. Variance Inflation Factor (VIF) and tolerance values were evaluated to ensure the empirical distinctiveness of the predictors.

Hypothesis testing was performed through mediation analysis using a path analysis approach grounded in the general linear model framework. This analysis employed observed variables computed from the total scores of each scale rather than latent variables. To address the issue of data non-normality, the significance of both direct and indirect effects was estimated using a bias-corrected bootstrapping procedure with 5,000 resamples. Bootstrapping was selected due to its robustness in generating accurate standard error estimates and confidence intervals for non-normally distributed data, without requiring assumptions of multivariate normality [47]. Because the structural model utilized observed rather than latent variables, global measurement model fit indices (e.g., CFI, TLI, RMSEA) for the full model are not reported, as this is standard practice in manifest-variable path analysis. Instead, measurement quality was established through the rigorous selection of previously validated instruments and the confirmation of strong internal consistency prior to computing the observed scores.

## Results

The present study comprised 200 adults (M age = 27.03 years, SD = 6.41). As indicated in Table 1, the sample was predominantly female (71%) and married (55%). With respect to weight status, the majority of participants were classified as Obesity Class I (41.5%) and Obesity Class II (42.5%), thereby substantiating the sample’s pertinence for the investigation of obesity-related psychological factors.

**Table 1:** Demographic Frequency.

	n	_%_
**Gender**		
**Male**	58	29%
**Female**	142	71%
**Marital Status**		
**Unmarried**	90	45%
**Married**	110	55%
**BMI criteria**		
**At Risk**	8	4%
**Obesity Class I**	83	41.5%
**Obesity Class II**	85	42.5%
**Obesity Class III**	24	12%

### Exploratory gender analysis

To address the gender imbalance in the sample and explore potential differences in the primary constructs, an exploratory analysis was conducted using the Mann-Whitney U test. The results indicated significant differences between men and women in self-compassion (U = 5039, p =.013) and self-love (U = 4911, p =.033). However, no significant gender differences were observed for self-acceptance (U = 4569, p =.224) or body image (U = 3801, p =.393).

The descriptive statistics for all study variables are presented in Table 2. The results of the normality tests (Shapiro–Wilk) indicated that the distributions for self-love, self-compassion, self-acceptance, and body image significantly deviated from normality (p < 0.05). Consequently, non-parametric Spearman correlations and bias-corrected bootstrapping methods were employed for subsequent analyses to ensure robust parameter estimation.

**Table 2:** Descriptive Statistics.

	Mean	Median	SD	IQR	Max	Min	Skewness	Kurtosis	Shapiro-Wilk
**SL**	42.79	44	8.06	13	63	23	-0.10	-0.66	.035
**SC**	38.45	38	4.54	4.3	58	26	0.52	2.51	<.001
**SA**	68.25	68	6.31	9	89	53	0.05	0.40	.031
**BI**	52.78	52	8.12	7	88	34	0.59	1.88	<.001

Abbreviations: SL, Self-Love; SC, Self- Compassion, SA, Self-Acceptance; BI, Body Image.

The bivariate associations among the study’s variables are presented in Table 3. The findings indicated a strong, significant positive association between self-compassion and self-acceptance (ρ = 0.472, p < 0.001). Additionally, body image demonstrated a moderate positive correlation with self-acceptance (ρ = 0.346, p < 0.001) and a modest positive association with self-love (ρ = 0.156, p < 0.05). However, the relationship between self-compassion and body image was not statistically significant (ρ = 0.125, p > 0.05). Similarly, self-love showed a non-significant correlation with self-compassion (ρ = 0.056, p > 0.05) and self-acceptance (ρ = 0.116, p > 0.05).

**Table 3:** Spearman Correlation Test.

	1	2	3	4
**1. Self-Love**	–			
**2. Self-Compassion**	.056	–		
**3. Body Image**	.156*	.125	–	
**4. Self-Acceptance**	.116	.472***	.346***	–

* p < .05, ** p < .01, *** p < .001

### Collinearity diagnostics

Prior to structural testing, potential multicollinearity was assessed to address conceptual overlap among the related constructs. The results indicated no evidence of multicollinearity, with tolerance values ranging from 0.917 to 0.958 and Variance Inflation Factor (VIF) values between 1.043 and 1.090, which are well below the commonly accepted cut-off threshold of VIF < 5. These findings establish the empirical distinctiveness of the predictors included in the regression models (Table 4).

**Table 4:** Regression Test Results (SL, SC → Body Image).

	*B (Unstd.)*	*SE*	*β (Std.)*	*t*	*p*
***(Intercept)***	26.61	6.444	–	4.129	<.001
**SL**	0.170	0.069	0.169	2.458	.015
**SC**	0.277	0.088	0.215	3.131	.002

Abbreviations: SL, Self-Love; SC, Self-Compassion.

R² = 0.083, Adj. R² = 0.073

RMSE = 7.817, F(2,197) = 8.884, p < .001

The linear regression analysis indicated that the model incorporating self-compassion and self-love simultaneously was significant in predicting body image, F(2,197) = 8.884, p < .001, accounting for 8.3% of the variance (R² = .083). Partially, self-compassion (β = .215, p = .002) and self-love (β = .169, p = .015) both emerged as significant positive predictors of body image. Notably, self-compassion demonstrated a stronger contribution than self-love. These findings indicate that individuals with higher levels of self-compassion and self-love tend to have a more positive body image, although the overall predictive strength of the model is relatively modest (Table 5).

**Table 5:** Regression Test Results (SL, SC, BI → Self-Acceptance).

	*B (Unstd.)*	*SE*	*β (Std.)*	*t*	*p*
***(Intercept)***	5.261	3.029	–	1.737	.084
**SL**	0.025	0.032	0.044	0.781	.436
**SC**	0.316	0.041	0.438	7.728	<.001
**BI**	0.201	0.032	0.359	6.241	<.001

Abbreviations: SL, Self-Love; SC, Self-Compassion; BI, Body Image.

R² = 0.406, Adj. R² = 0.397

RMSE = 3.525, F(3,196) = 44.75, p < .001

The multiple regression analysis revealed that the model incorporating self-compassion, self-love, and body image simultaneously significantly predicted self-acceptance, F(3,196) = 44.75, p < .001, explaining 40.6% of the variance (R² = .406). Partially, self-compassion (β = .438, p < .001) and body image (β = .359, p < .001) were significant positive predictors of self-acceptance, with self-compassion functioning as the strongest predictor. Conversely, self-love did not demonstrate a significant effect (β = .044, p = .436). These findings suggest that self-acceptance is predominantly influenced by an individual’s capacity to exhibit self-compassion and maintain a positive perception of their body, rather than by general self-evaluation.

Finally, mediation analysis (Table 6) was conducted to test whether body image mediated the pathways from self-love or self-compassion to self-acceptance. The analysis indicated that the indirect effects for both self-love (β = 0.088, 95% CI [0.019, 0.177]) and self-compassion (β = 0.086, 95% CI [0.019, 0.176]) were significant, as the confidence intervals did not include zero. The findings suggest that body image partially mediates the relationship between self-compassion and self-acceptance, and fully mediates the relationship between self-love and self-acceptance. A comprehensive summary of the hypothesis testing results is provided in the supplementary material (S1 Table).

**Table 6:** Mediation Test Results.

	*Estimate* (β)	*SE*	95% CI	*p-value*
**Total Effect (SL → SA)**	0.161	0.072	[0,010, 0,293]	.024
**Direct Effect (SL → SA)**	0.074	0.057	[-0.038, 0.185]	.196
**Indirect Effect (SL → BI → SA)**	0.088	0.041	[0.019, 0.177]	.032
**a1 path (SL → BI)**	0.193	0.069	[0.044, 0.315]	.005
**b path (BI → SA)**	0.455	0.077	[0.297, 0.598]	<.001
**Total Effect (SC → SA)**	0.527	0.054	[0.409, 0.621]	<.001
**Direct Effect (SC → SA)**	0.441	0.054	[0.335, 0.543]	<.001
**Indirect Effect (SC → BI → SA)**	0.086	0.039	[0.019, 0.176]	.029
**a2 path (SC → BI)**	0.234	0.086	[0.053, 0.390]	.007

Abbreviations: SL, Self-Love; SC, Self- Compassion, SA, Self-Acceptance; BI, Body Image.

## Discussion

The present study aimed to elucidate the psychological mechanisms linking self-compassion, self-love, and body image to self-acceptance among 200 Indonesian adults with obesity. The final structural model accounted for a substantial 40.6% of the variance in self-acceptance, demonstrating the high practical significance of these internal resources in populations facing chronic weight stigma. Self-compassion emerged as the strongest predictor of self-acceptance (β =.438, p <.001), exerting both a direct influence and an indirect effect via body image, indicating partial mediation. In contrast, self-love influenced self-acceptance exclusively through the enhancement of body image, indicating full mediation. Additionally, exploratory analyses revealed that men reported higher levels of self-compassion and self-love than women, adding necessary nuance to how these protective constructs operate across genders in understudied populations.

Crucially, to address potential concerns regarding conceptual overlap, rigorous collinearity diagnostics confirmed that self-compassion and self-acceptance are empirically distinct constructs, despite their strong bivariate correlation (ρ =.472). The exceptionally low VIF values observed here (ranging from 1.043 to 1.090) indicate that these constructs share less than 10% of their variance due to collinearity, confirming minimal measurement redundancy, as VIF values below 1.5 are generally recognized as indicating minimal multicollinearity [47]. This empirical separability aligns directly with robust factor-analytic evidence showing that self-compassion and self-acceptance load onto distinct factors rather than a unidimensional construct [24–26]. Theoretically, this distinction is grounded in their differing psychological functions: self-compassion operates as an active, tripartite emotion-regulation strategy utilized during moments of distress [19], whereas self-acceptance is a stable, cognitive appraisal of inherent worth independent of physical attributes or external outcomes [51]. Prior correlational and intervention studies consistently support this discriminant validity through divergent predictive patterns on outcomes such as anxiety, self-blame, and life satisfaction [27,28]. The distinct mediation pathways observed in the present model provide further structural evidence for this separation; self-compassion’s robust direct effect highlights its capacity to foster psychological well-being independent of specific body evaluations, whereas self-love requires the proximal modification of body image to achieve global self-acceptance.

The finding that body image functions as a significant mediator aligns with both theoretical frameworks and intervention research emphasizing the reduction of body-related shame and self-criticism in populations with obesity [52,53]. Structural and interventional models have consistently demonstrated that compassion-focused approaches attenuate internalized weight stigma, emotional eating, and body dissatisfaction, which subsequently improves health-related outcomes and adaptive behavior [52,54,55]. The present study extends this literature by elucidating distinct mediational pathways for different self-related constructs. Specifically, body image fully mediated the relationship between self-love and self-acceptance (indirect effect β =.088, 95% CI [0.019, 0.177]), indicating that self-love operates exclusively through the proximal modification of body-image-related evaluations to influence global self-worth in this stigmatized sample [52,53]. In contrast, body image only partially mediated the relationship between self-compassion and self-acceptance (indirect effect β =.086, 95% CI [0.019, 0.176]), as self-compassion maintained a robust direct association (β =.441, p <.001). This dual-pathway pattern suggests that while self-love is tightly coupled to body-specific evaluations, self-compassion operates through broader regulatory functions—such as generalized emotion regulation and cognitive reappraisal—fostering psychological well-being across multiple life domains independent of body image [54, 56].

Beyond specific structural pathways, the overall predictive capacity of the model underscores the profound practical impact of these internal resources. The final regression model accounted for 40.6% of the variance in self-acceptance (Adjusted R² =.397). According to Cohen’s conventional benchmarks—which classify R² values of.02,.13, and.26 as small, medium, and large effect sizes, respectively [57] —the observed R² substantially exceeds the threshold for a large effect. This indicates that the integration of self-compassion, body image, and self-love explains a highly meaningful proportion of individual differences in self-acceptance among Indonesian adults with obesity. When contextualized within the broader literature, this explanatory power is comparable to, or exceeds, effect sizes reported in similar psychosocial models. For instance, while specific proximal predictors like family fat talk have been shown to explain up to 62.6% of the variance in narrower outcomes such as body dissatisfaction [58], structural equation models examining global self-acceptance in overweight samples rarely account for such a robust proportion of total variance [53].

Theoretically, this substantial effect size reinforces models that position self-compassion and body image as central determinants of psychological well-being in stigmatized populations. Practically, it suggests that clinical interventions targeting self-compassion and body-related perceptions have the potential to produce clinically meaningful improvements in overall self-acceptance. Nevertheless, the model’s R² also reveals that approximately 59% of the variance remains unexplained. This serves as an important reminder that global self-acceptance is a complex, multidimensional construct. Consequently, future comprehensive interventions must complement individual-level psychological strategies by addressing additional systemic and contextual factors, including social support, weight stigma experiences, socioeconomic status, and broader cultural values.

In response to the necessity of exploring demographic variations, the exploratory gender analyses identified significant differences in specific internal resources, with men reporting higher levels of both self-compassion and self-love than women. This pattern aligns with meta-analytic evidence across general adult populations, which similarly demonstrates a small but consistent male advantage in self-compassion [59]. Researchers suggest that socialization toward traditional masculine role orientations—which often emphasize independence and self-reliance—may inadvertently facilitate more compassionate self-responding and self-prioritization [60]. The emergence of this pattern in the current sample suggests that these gendered socialization processes regarding self-compassion and self-love may operate similarly within the Indonesian cultural context as they do in the predominantly Western samples previously studied.

Interestingly, despite these disparities in internal regulatory resources, no significant gender differences emerged for global self-acceptance or body image. This divergence from established obesity literature—which typically reports significantly higher weight bias internalization and body-shape concerns among women—warrants careful interpretation. Methodologically, the predominantly female sample (71%) may have constrained the statistical power required to detect subtle differences, and the specific multidimensional body image scale utilized here may capture different evaluative facets than standard weight bias measures [61,62]. Culturally, weight-related distress in Asian contexts often manifests through collectivistic expectations and family-level teasing, which may exert unique, cross-gender pressures distinct from Western normative standards [58,63]. Consequently, men and women in this population may arrive at similar baseline levels of body image and self-acceptance through divergent psychological pathways. For instance, women might compensate for lower self-compassion by leveraging unmeasured external protective factors, such as religious coping or family cohesion [64], whereas men’s higher self-compassion may be offset by masculine norms that discourage the acknowledgment of body-related vulnerability [61]. Ultimately, these nuances highlight the necessity of developing gender-sensitive clinical interventions that account for these differential psychosocial pathways to self-acceptance among adults with obesity.

The structural pathways observed in the present study must be interpreted within the specific sociocultural framework of Indonesia, as the manifestation, magnitude, and primary sources of weight stigma vary significantly across cultural contexts. In contrast to Western individualistic societies—where obesity is frequently attributed to internal, personal moral failures (e.g., lack of willpower) and stigma often originates from broader societal or peer levels [65] weight-related distress in collectivistic Asian cultures is profoundly shaped by familial dynamics. In these interdependent contexts, body size is often perceived as a reflection of the family or social group rather than merely the autonomous individual [66]. Consequently, family-level weight stigma, such as parental teasing or pervasive “fat talk” during everyday familial interactions, represents a uniquely potent source of psychological harm. Research among Chinese Indonesian women, for example, demonstrated that family fat talk accounted for a staggering 62.6% of the variance in body dissatisfaction [58], and qualitative analyses of Indonesian communities consistently highlight family members as the primary sources of fat-shaming narratives [67]. This robust familial influence frequently exceeds the impact of peer-based teasing commonly observed in Western samples, underscoring how collectivistic norms distinctly shape the internalization of weight bias [65].

Viewed through this cultural lens, the strong associations identified in the current model—particularly the robust link between self-compassion and self-acceptance (ρ =.472)—take on added contextual significance. In an environment where external, family-based weight criticism is culturally normalized and overt self-love may occasionally conflict with social norms of modesty, self-compassion offers a culturally congruent internal buffer. It allows individuals to maintain global self-worth despite intense, unavoidable pressures to conform to familial weight expectations [67]. Furthermore, the partial mediation of body image in the self-compassion pathway suggests that this internal resource mitigates distress through both body-dependent and body-independent regulatory processes. This dual capability is particularly adaptive in collectivistic contexts where family-level stigma may directly undermine global self-worth irrespective of an individual’s personal body image improvements [58]. However, in alignment with rigorous methodological boundaries, it is imperative to reiterate that these cultural dynamics serve strictly as an interpretive framework for the current findings. Future research must explicitly operationalize and measure cultural dimensions—such as interdependent self-construal, family-level weight stigma, and specific collectivistic values—to empirically test these culturally embedded mediation pathways among Indonesian populations with obesity.

Translating the structural pathways identified in this study into clinical practice aligns with a robust and growing evidence base supporting self-compassion interventions for populations with obesity. Randomized controlled trials and pilot programs—such as the acceptance-, mindfulness-, and compassion-based Kg-Free program—demonstrate that these targeted interventions effectively reduce internalized weight stigma, shame, and body dissatisfaction while promoting adaptive eating behaviors [52,54]. Notably, large-scale trials supplementing standard behavioral weight-management programs with self-compassion modules reveal that, while immediate additional weight loss may not always be achieved, participants reliably experience significant psychological benefits, including heightened self-kindness and reduced self-judgment [56]. Consistent with systematic reviews, these findings indicate that cultivating self-compassion yields meaningful clinical improvements in body image and related distress [32]. Consequently, clinical goals in obesity management must be broadened; clinicians should monitor psychological mediators such as shame and self-criticism as primary change targets, communicating to patients that psychological resilience represents a deeply meaningful clinical outcome independent of immediate weight reduction.

Within the Indonesian healthcare infrastructure, these mechanistic findings have direct and urgent implications for community health centers (Puskesmas) and integrated health posts (Posyandu), which serve as the primary grassroots platforms for obesity management [68]. Integrating compassion training into these local behavioral programs could be highly effective, provided the interventions are culturally adapted to address the unique manifestations of weight stigma in Indonesia. Because family-level “fat talk” and collectivistic pressures are primary sources of body-image distress [58,67], interventions must explicitly equip individuals with skills to respond compassionately to family-based criticism. Group-based compassion interventions are particularly well-suited for this collectivistic society, as they leverage the core component of common humanity to reduce stigma-induced isolation. Furthermore, these programs could be significantly enhanced by incorporating family psychoeducation to reduce domestic weight stigma, and by integrating culturally resonant religious or spiritual frameworks—such as Islamic concepts of self-mercy and forgiveness or Buddhist principles of loving-kindness—to make self-compassion practices more conceptually accessible. Future pilot testing of such culturally adapted, community-based self-compassion interventions in Puskesmas and Posyandu settings is critical to establishing their feasibility, acceptability, and efficacy for this understudied population.

### Strength and limitation

Despite the theoretical and practical contributions of this study, several limitations must be rigorously acknowledged. Foremost, the cross-sectional design precludes any definitive causal inferences regarding the directionality of the observed relationships. Although the mediation models position self-compassion and body image as proximal predictors of self-acceptance [52,53], these structural associations remain correlational and may well be bidirectional. For instance, individuals with higher baseline self-acceptance might naturally engage in more self-compassionate responding and maintain a positive body image, thereby creating mutually reinforcing cycles of psychological well-being [69]. To establish true temporal precedence and definitively test these causal pathways, future research must employ longitudinal designs or experimental interventions that manipulate self-compassion to observe downstream effects on body image and global self-acceptance.

Methodologically, the reliance on self-report questionnaires introduces vulnerabilities related to shared method variance and social desirability bias [70]. A specific manifestation of this vulnerability is the use of self-reported height and weight to calculate Body Mass Index (BMI). Extensive research documents that self-reported BMI frequently underestimates true BMI due to the systematic overreporting of height and underreporting of weight, particularly among individuals with higher actual body mass [71]. While this underestimation suggests the current sample comprises individuals willing to acknowledge their weight status, it introduces the risk of categorical misclassification around the Asia-Pacific inclusion thresholds. Future studies must utilize objective anthropometric measurements to guarantee diagnostic accuracy, and could robustly complement traditional survey methods by incorporating implicit or behavioral indicators of self-acceptance and self-compassion.

Demographically, the sample’s composition restricts the broader generalizability of the findings. The cohort was predominantly female (71%), a common imbalance in obesity research reflecting differential help-seeking behaviors and study engagement. While our exploratory analyses provided preliminary insights into male experiences, the unequal group sizes limit statistical power to detect complex gender-by-treatment interaction effects or finely stratified pathways [61]. Additionally, the sample was restricted to younger adults (aged 18–40 years). Because emotion regulation, appearance-based self-worth, and weight-stigma experiences evolve dynamically across the lifespan, these findings may not fully generalize to older populations with obesity, who often face distinct health complications and psychosocial challenges [72]. Future investigations should employ targeted, stratified recruitment to ensure balanced gender representation and examine developmental moderation across different life stages.

Finally, the current model did not account for several potentially influential confounding variables. Crucially, as highlighted in the preceding cross-cultural discussion, specific cultural dimensions—such as interdependent self-construal, collectivistic values, and family-level weight stigma—were not empirically measured. Moreover, the study did not assess clinical and historical variables, including the duration of obesity, presence of medical comorbidities, weight-loss history, or explicit treatment-seeking behaviors. The absence of these variables, coupled with a convenience sampling approach within specific Indonesian regions, limits the ability to translate these findings into highly individualized clinical recommendations. Addressing these unmeasured contextual and clinical factors remains a critical frontier for comprehensively mapping the psychological landscape of obesity in non-Western populations.

## Conclusion

This study provides novel evidence regarding the structural mechanisms linking self-compassion, self-love, and body image to global self-acceptance among Indonesian adults with obesity. Crucially, the findings confirm that self-compassion and self-acceptance are empirically distinct constructs (VIF < 1.1) despite their strong correlation, supporting theoretical models that differentiate active emotion-regulation processes from stable cognitive appraisals of self-worth. Furthermore, the mediational analyses revealed distinct pathways: body image fully mediated the relationship between self-love and self-acceptance, while only partially mediating the relationship between self-compassion and self-acceptance. This dual-pathway model indicates that self-compassion operates through both body-image-dependent and body-image-independent structural routes.

The substantial explanatory power of the final model (R² = .406) demonstrates that these internal resources account for a highly meaningful proportion of the variance in self-acceptance. Additionally, exploratory analyses added critical demographic nuance, revealing higher levels of self-compassion and self-love among men, which underscores the need for gender-sensitive approaches. Practically, these findings strongly support the integration of self-compassion training into standard behavioral weight management programs within Indonesian primary healthcare settings (e.g., *Puskesmas* and *Posyandu*) to effectively reduce internalized stigma and shame. Acknowledging the limitations of the current cross-sectional design, future research must employ longitudinal or experimental methodologies to definitively establish causal precedence. Subsequent studies should also incorporate objective anthropometric measurements, ensure balanced gender representation, and explicitly measure culturally specific dynamics—such as family-level weight stigma and interdependent self-construal—to further advance our understanding of psychological well-being in this understudied population.

## Supporting information

S1 Table Summary of hypothesis testing results. (DOCX)
