# Supplementary material for: A path toward self-acceptance: The mediating effects of body image between self-love, self-compassion, and self-acceptance in people with obesity
Source: PLOS Ment Health. 2026 May 19;3(5):e0000609. doi: 10.1371/journal.pmen.0000609 (PMC13186385; doi:10.1371/journal.pmen.0000609)
Supplement: S1 Table — (DOCX) [file pmen.0000609.s001.docx]

**Supplementary Materials**

**Table S1. Summary of Hypothesis Testing Results**

| Hypotheses Proposed | Results |
| --- | --- |
| H1: Higher levels of self-love are positively associated with greater self-acceptance among individuals with obesity in Indonesia. | Not Supported |
| H2: Higher levels of self-compassion are positively associated with greater self-acceptance among individuals with obesity in Indonesia. | Supported |
| H3: A more positive body image is positively associated with greater self-acceptance among individuals with obesity in Indonesia. | Not Supported |
| H4: Higher levels of self-love are positively associated with a more positive body image among individuals with obesity in Indonesia. | Supported |
| H5: Higher levels of self-compassion are positively associated with a more positive body image among individuals with obesity in Indonesia. | Supported |
| H6: Body image mediates the relationship between self-love and self-acceptance among individuals with obesity in Indonesia. | Not Supported |
| H7: Body image mediates the relationship between self-compassion and self-acceptance among individuals with obesity in Indonesia. | Not Supported |
